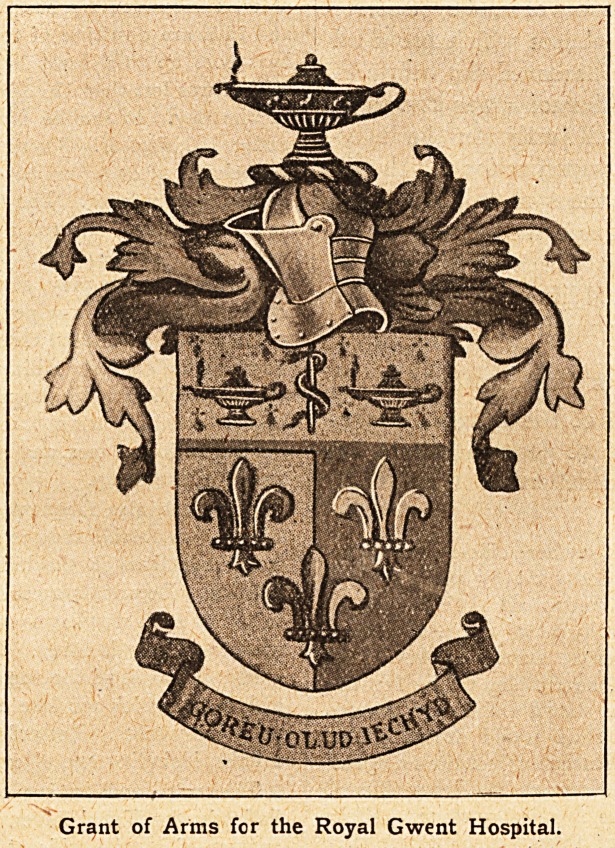# Royal Gwent Hospital

**Published:** 1919-08-30

**Authors:** 


### ROYAL GWENT HOSPITAL.

### Grant of Arms.

Thanks to Sir Garrod Thomas, the Royal Gwent Hospital has been given a Grant of Arms, for use on documents and seals, etc. The award of the Grant is in the following terms :

"To all and singular to whom these presents shall come. Henry Farnham Burke, Esq., Companion of the Most Honourable Order of the Bath, Commander of the Royal Victorian Order, Garter Principal King of Arms; William Hy. Weldon, Esq., C.V.O., Clarenceux King of Arms, and Harold Athill, Member of the Royal Victorian Order, Norroy King of Arms, send greeting. Whereas Sir Abraham Garrod Thomas, Knight, Chairman of the Board of Directors of the Royal Gwent Hospital, hath represented unto the Right Hon. Edmund Bernard Talbot, Companion of the Distinguished Service Order, Member of the Royal Victorian Order, and Deputy of the Most Honourable Bernard Marmaduke, Duke of Norfolk, Earl Marshal and Hereditary Marshal of England, that in July, 1913, his Majesty was graciously pleased to signify his approval of the Newport and Monmouthshire Hospital being named the Royal Gwent Hospital, and the Directors of the said Hospital being desirous that armorial bearings should be used on the seals made use of by them as Directors of and on behalf of the said hospital, therefore request the favour of his Lordship's warrant for an order granting and assigning the said armorial ensigns as may be proper to be approved and used by them and their successors, Directors of the Royal Gwent Hospital on seals, shields, banners, or otherwise, according to the laws of Arms; and forasmuch as his Lordship did by warrant in his hand, and the seal of the Earl Marshal bearing the date eleventh day of November last authorise and direct us to grant and assign the said armorial ensigns; accordingly know ye therefore, that the said Garter, Clarenceux, and Norroy, in pursuance of his Lordship's warrant and by virtue of the laws patent of our several offices, to each of us respectively granted, do by these presents grant and assign unto the Directors of the Royal Gwent Hospital the arms following, that is to say, per pale azure and sable, three fleur de lys, or on a chief ermine the Rod of Æsculapius between two antique lamps inflamed of the third, and for the crest on a wreath of the colours an antique lamp inflamed; gates, charged with three fleur de lys in fess, or, as the same are in the margin hereof, the same plainly depicted to be borne and used for ever hereafter by the Directors of the Royal Gwent Hospital and their successors on seals, shields, banners, or otherwise, according to the laws of arms.

Grant of Arms for the Royal Gwent Hospital.

"For witness whereof, we, the said Garter, Clarenceux, and Norroy, Kings of Arms, have to these presents subscribed our names, and fixed the seals of our several offices this 24th day of January, in the ninth year of the reign of our Sovereign Lord, King George the Fifth, by the Grace of God, in the United Kingdom of Great Britain and Ireland and of the British Dominions beyond the Seas, King, Defender of the Faith, etc., and in the year of our Lord 1919."

Then follow the signatures of the Kings of Arms.

The fleur de lys has been taken from the arms of the old Kings of Gwent; the rod of Æsculapius represents medicine, and the lamp symbolises nursing. Longfellow's poem, "Santa Filomena," spoke of Florence Nightingale as "the lady with the lamp." The motto, "Goreu Olud Iechyd," is Welsh for "Best Wealth—Health."

## Figures and Tables

**Figure f1:**